# Targeted Testing and Treatment To Reduce Human Malaria Transmission in High-Risk Populations: A Systematic Review

**DOI:** 10.4269/ajtmh.23-0097

**Published:** 2024-03-12

**Authors:** Beena Bhamani, Elisabet Martí Coma-Cros, Maria Tusell, Vita Mithi, Elisa Serra-Casas, Nana Aba Williams, Kim A. Lindblade, Koya C. Allen

**Affiliations:** ^1^ Barcelona Institute for Global Health (ISGlobal), Hospital Clínic, University of Barcelona, Barcelona, Spain;; ^2^ Armref Data for Action in Public Health Research Consultancy, Mzuzu, Malawi;; ^3^ Society for Research on Nicotine and Tobacco—Genetics and Omics Network, Madison, Wisconsin;; ^4^ Leaders of Africa Institute, Baltimore, Maryland;; ^5^ Global Malaria Programme, World Health Organization, Geneva, Switzerland

## Abstract

As countries approach elimination of malaria, groups with increased exposure to malaria vectors or poor access to health services may serve as important human reservoirs of infection that help maintain transmission in the community. Parasitological testing and treatment targeted to these groups may reduce malaria transmission overall. This systematic review assessed the effectiveness of targeted testing and treatment (TTaT) to reduce malaria transmission, the contextual factors, and the results of modeling studies that estimated the intervention’s potential impact. Bibliographic searches were conducted in March 2021 and updated in April 2022, and a total of 1,210 articles were identified. Three studies were included for outcome data: one factorial cluster randomized controlled trial (cRCT) in Kenya (5,233 participants), one cRCT in Ghana (3,046 participants), and one controlled before-and-after cohort study in schoolchildren in Malawi (786 participants). Nine reports were included for contextual factors, and two were included for mathematical modeling. Data on outcomes from the three studies suggested that at the community level, TTaT would result in little to no difference in the incidence of malaria infection (measured via active surveillance), adverse events, and severe AEs. In contrast, the effects of TTaT on prevalence (malaria parasitemia) among those targeted by the intervention were found to include a short-term impact on reducing transmission but little to no impact on transmission for extended periods. Future iterations of this review should ensure consideration for populations proven to host the vast majority of the reservoir of infection in lower-transmission settings to determine the effectiveness of the intervention.

## INTRODUCTION

As malaria transmission declines and approaches zero, evidence shows that the infection tends to cluster geographically (i.e., households, neighborhoods) or socially (i.e., common occupations, shared travel) within populations.^1^^,^^2^ A reasonable approach to fight infections clustered in subgroups of the population, when people at a high risk of infection can be easily identified, is targeted testing and treatment (TTaT). Targeted testing and treatment is an active case detection^3^ strategy that includes parasitologic testing (with or without prior symptom screening), followed by treatment of confirmed cases with a full therapeutic course of an antimalarial medicine, including radical treatment of infections caused by *Plasmodium vivax* and *Plasmodium ovale*. The populations targeted by the TTaT intervention include high-risk individuals defined based on demographic, occupational, and/or exposure characteristics and considered to comprise a large proportion of the parasite reservoir of infection in an area with *Plasmodium *spp. affecting humans.^3^ For TTaT, identifying these high-risk groups may serve as an indicator for capturing malaria cases that need to be successfully treated both for their own individual benefit and to reduce community transmission of malaria.

This systematic review served to collate the current evidence to further understand how populations at high risk, and surrounding populations, could benefit from TTaT to progress towards malaria control and elimination.^3^ In particular, we aimed to determine the benefits and harms of TTaT in adults and children at higher risk for malaria infection in areas with ongoing transmission or malariogenic potential. In addition, we assessed the factors that might modify the effects of TTaT in high-risk populations^3^ and identify evidence on contextual factors (values and preferences, health equity, resource use, acceptability, and feasibility) and mathematical modeling.

## MATERIALS AND METHODS

This systematic review followed the Preferred Reporting Items for Systematic Reviews and Meta-Analyses (PRISMA) format, and the protocol was registered in the International Prospective Register of Systematic Reviews (PROSPERO; CRD42021232451).^4^

Complete details of the eligibility criteria, search strategy, study selection, data collection, and analysis have been described elsewhere.^5^ An overview of the methods is provided below.

### Population, intervention, comparison, and outcomes (PICO).

This review aimed to answer the following question “What are the relative effects (benefits and harms) of TTaT compared to no TTaT in adults and children at increased risk of malaria infection?” The targeted population was specifically those adults and children defined as being at high risk based on demographics (e.g., age, gender, social characteristics), occupation (e.g., agriculture workers, military personnel, miners, forest-goers, peacekeepers), or other exposure characteristics (e.g., outdoor activities, migration). Particular emphasis was given to studies that targeted vulnerable populations serving as parasite reservoirs and contributing to continuous transmission if not timely tested, treated, and tracked.^6^^,^^7^ For this review, the TTaT strategy was defined as parasitologic testing (with or without prior symptom screening) for malaria infection with *Plasmodium* spp. affecting humans, followed by treatment of confirmed cases with a full therapeutic course of antimalarial medicine, including treatment of liver-stage parasites for infections caused by *P. vivax* and *P. ovale*.^3^ Interventions that were implemented as a part of other disease strategies, such as integrated community case management or community health worker strategies, were eligible for inclusion if the TTaT intervention was retained in its entirety. The control arm was no implementation of TTaT.

### Selection of studies.

Studies included in this review assessed at least one main outcome at the community level or among the targeted population: incidence of malaria infection (through active surveillance) at the community level, prevalence of malaria infection (point prevalence of malaria parasitemia) at the community level, elimination (defined as zero indigenous or local cases for a period during the transmission season), incidence of clinical malaria (passive surveillance/passive case detection/incidence in cohort), or number of indigenous malaria cases at the community level, drug resistance at the community level, adverse events (AEs) among the group targeted by the intervention, and prevalence of infection among the group targeted by the intervention.

### Data extraction, analysis, and assessment of quality.

The detailed selection strategy and selected keywords used are presented in Supplemental Table 1. The final search strategy for the review was developed in collaboration with a systematic review information specialist. The final search was conducted in March 2021 and was updated in April 2022. For the study selection process, each study was reviewed by two authors independently for eligibility and quality. All studies identified as potentially eligible based on the title and abstract were retrieved for a full-text review and were reviewed by two authors independently. The detailed methods on data extraction and synthesis, risk of bias assessments in individual studies, assessment of heterogeneity, and quality are described elsewhere.^5^

## RESULTS

### Study selection.

**Figure 1.**
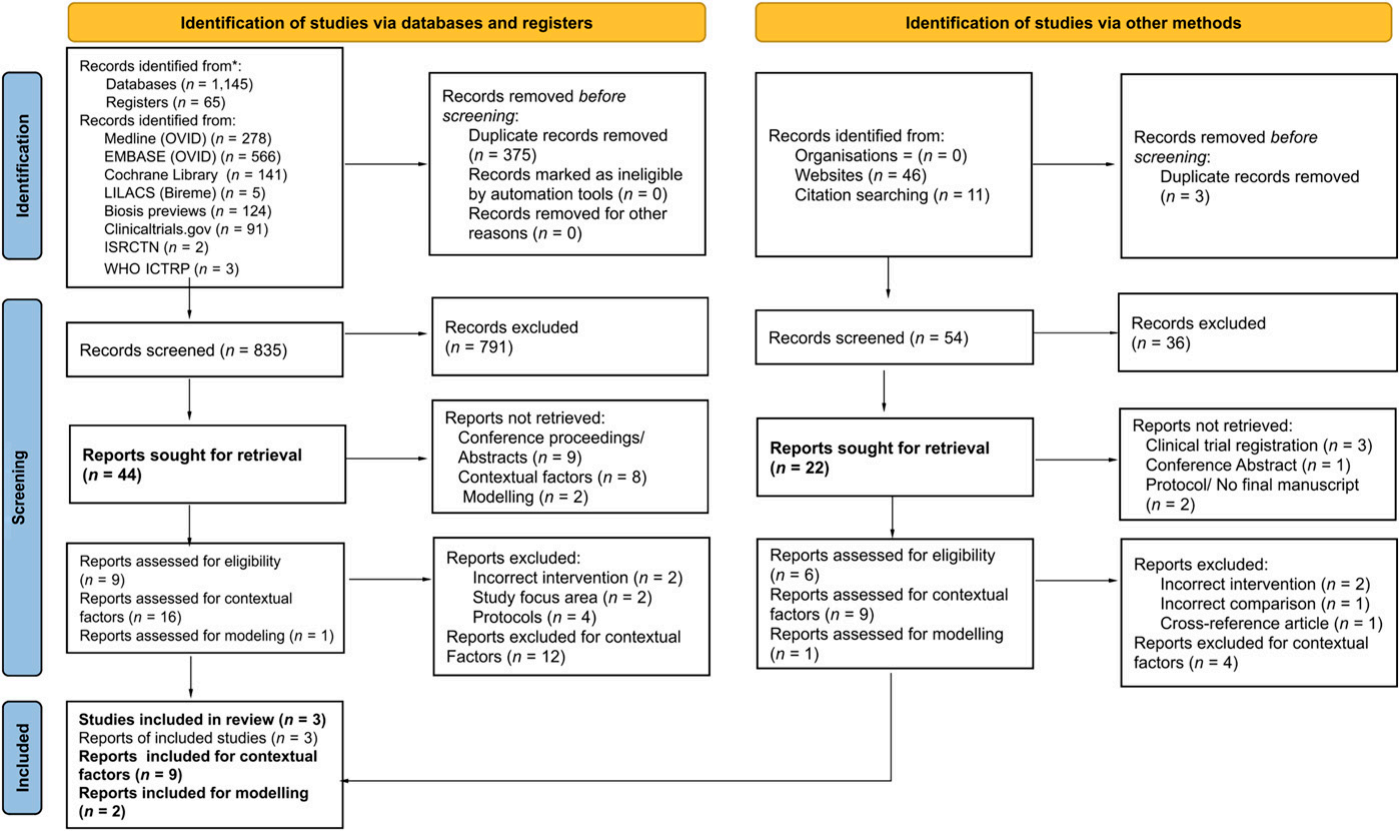
Preferred Reporting Items for Systematic Reviews and Meta-Analyses (PRISMA) diagram.

The search strategy yielded 1,210 records (1,145 from databases and 65 from registers), and 57 additional records were identified from other methods. Of these, 378 duplicates were removed before screening. A total of 889 records were reviewed for inclusion, and 827 were excluded at the title and abstract screening stage. Of the remaining 66 records eligible for full-text screening, 25 could not be retrieved because they were conference proceedings, abstracts, or trial registrations. A total of 41 reports underwent full-text review for eligibility: 15 reports were assessed for outcome data, 25 reports were assessed for inclusion of contextual factors, and one report was assessed for mathematical modeling. After full-text screening, three studies were included in the review for outcomes, nine reports were included for contextual factors, and two studies were included for modeling, of which one was already included for outcomes. The detailed PRISMA flow diagram is presented in Figure 1.

A total of 28 records were excluded at the full-text screening stage for the following reasons: incorrect intervention (4), incorrect comparison (1), incorrect study focus area (2), cross-referenced articles (1), study protocols (4), and no assessment of the contextual factors of interest (16). All studies that did not meet eligibility criteria at the full-text stage are listed with their reasons for exclusion in Supplemental Table 2.

### Included studies.

Three studies were eligible for inclusion in the review: one factorial cluster randomized controlled trial (cRCT) in Kenya by Halliday et al.,^8^ one cRCT in Ghana by Baiden et al.,^9^ and one controlled before-and-after (cBAF) cohort study in schoolchildren in Malawi by Cohee et al.^10^ Characteristics of included studies are provided in Table 1. A total of nine reports were included for contextual factors,^11^^–^^19^ and two modeling studies were included.^10^^,^^20^

**Table 1:** Characteristics of included studies

Study	Location	Year(s)	Study Design	Intervention	Outcomes Reported
Halliday et al.^8^	Southern Coast, Kenya	2010–2012	Factorial cRCT	Target population: school children 5–20 years old Number of study participants: 5,233 Intervention: screening and treatment Comparator: no intervention Screening method: RDT and microscopy Drug: AL Time/rounds: 5 rounds (2 rounds after 3 months, and 2 rounds after 6 months)	Prevalence of infection among those targeted at 12 months postintervention Prevalence of infection among those targeted at 24 months postintervention AEs (24–48 hours posttreatment to 28 days) SAEs (for 24 months follow-up)
Baiden et al.^9^	Forest-savannah transition zone, Ghana	2009–2012	cRCT	Target population: children < 24 months Number of study participants: 3,046 Intervention: RDT-based test & treatment Comparator: standard of care/clinical judgement Screening method: RDT Drug: ACTs Time/rounds: 2 years from first febrile episode	Incidence of malaria infection at the community level (24 months follow-up period) SAEs (24 months follow-up period)
Cohee et al.^10^	Southern Malawi	2015	cBAF/cohort	Target population: schoolchildren 5–15 years old Number of study participants: 786 Intervention: screening and treatment Comparator: no intervention Screening method: RDT and PCR Drug: AL Rounds: 3 rounds at 1, 2, and 6 weeks	Prevalence of infection among those targeted (6 weeks postintervention)

ACTs = artemisinin-based combination treatments; AEs = adverse events; AL = artemether-lumefantrine; cBAF = controlled before and after; cRCT = cluster randomized controlled trial; SAEs = serious adverse events; RDT = rapid diagnostic test; PCR = polymerase chain reaction; TTaT = targeted testing and treatment.

Halliday et al.^8^ conducted a factorial cRCT in 5,233 schoolchildren in 101 schools in the Kwale and Msambweni districts on the southern coast of Kenya between 2010 and 2012. The trial aimed to determine the effects of intermittent screening and treatment (IST) for malaria on health, sustained attention, and education in low to moderate malaria transmission settings, as well as the impact of a literacy intervention on education. During the IST, children were screened once per school term for malaria parasitemia using a rapid diagnostic test (RDT), and children (with or without malaria symptoms) found to be RDT positive were treated with a six-dose regimen of artemether-lumefantrine (AL) over 3 days. Five rounds of screening and treatment were implemented.^8^ In a cRCT, Baiden et al.^9^ hypothesized that the effects of a test-based malaria control strategy would increase the incidence of malaria and concurrently anemia while decreasing the use of artemisinin-based combination therapies (ACTs) based on the increased accuracy of treatment in comparison with that of malaria diagnosis using clinical judgement. This hypothesis was made considering the revised malaria treatment guidelines issued by the WHO in 2010 to reduce the unwarranted use of ACTs in children by restricting them to only positive RDT cases. However, this shift of approach was argued with the hypothesis that restricting ACTs to only RDT-positive cases would remove the protective effect of ACTs in children with febrile illnesses, thus increasing malaria incidence and anemia in children.^9^ The study,^9^ conducted in six districts that lay within the forest-savannah transition zone of perennially high malaria transmission in Ghana, was implemented between February 2009 and July 2012. Children participating in the intervention who reported fever were tested with an RDT and received ACTs when positive. Children in comparison groups were assessed for malaria using only clinical management of febrile illness presentation at health centers.^9^ In a cBAF cohort study, Cohee et al.^10^ also targeted schoolchildren, which was thought to be an important reservoir of infection in southern Malawi. A total of 786 students across four schools were tested and, if positive, treated with AL.

Among the key outcomes assessed in this review, the incidence of malaria infection was assessed at the community level by RDT and microscopy by Baiden et al.^9^ during the 24-month follow-up period after the first episode of febrile illness in children enrolled in June 2010 and monitored until June 2012. Adverse events were measured in the group targeted by the intervention by using active surveillance in a study by Halliday et al.^8^ Serious AEs (SAEs) among the group targeted by the intervention (categorized as mortality) were assessed during the 24 months of follow-up in studies by Halliday et al.^8^ and Baiden et al.^9^ The prevalence of malaria infection among the group targeted by the intervention was measured by Halliday et al.^8^ at a mean follow-up of 12 and 24 months. The prevalence of malaria infection was also measured by Cohee et al.^10^ at a mean follow-up of 6 weeks.

### Assessment of quality.

Assessments for risk of bias for outcomes in the two cRCTs by Baiden et al.^9^ and Halliday et al.^8^ are summarized in Supplemental Figure 1 for overall risk by domain and as percentages for intention to treat analyses for each domain category.

Both studies^8^^,^^9^ were rated as having low risk of bias for the randomization process, recruitment of participants, deviations for outcome, and measurement of outcomes. For all outcomes except AEs reported by Halliday et al.,^8^ missing outcome data were also rated as having low risk of bias. In the report by Halliday et al.,^8^ there were some concerns regarding AEs due to a lack of reporting in the control arm of the trial. Likewise, the risk of bias for selection of the reported result was rated as low for all outcomes by Halliday et al.,^8^ since reported primary and secondary outcomes were the same as those planned in the trial protocols and registration. However, for Baiden et al.,^9^ selection of the reported result was ranked as having a high risk of bias because although the reported outcome was consistent with the trial registration to assess the incidence of malaria, these authors reported additional outcomes, including episodes of malaria, which accounted for repeated illnesses, but did not assess the number of children in the intervention and control arms that had malaria overall. Incidence was instead categorized by all episodes, episodes after first fever, and repeated malaria. Crude prevalence or number of clinical cases was not reported. In addition, the researchers conducted a multilevel Poisson regression analysis to calculate incidence and incidence rate ratios (IRR) for comparison in study arms but did not perform a generalized model accounting for potential demographics and confounders to assess the risk of malaria infection in study arms.

Supplemental Figure 2 summarizes the risk of bias assessment for the cBAF cohort study by Cohee et al.^10^ All domains were rated as having a low risk of bias except for bias in selection of the reported result. The risk of bias for selection of the reported result was moderate because there were multiple measurements and analyses used within the outcome domain. The contribution to transmission was evaluated in four ways, namely, 1) gametocyte prevalence, which was defined as the proportion of participants with any gametocytes, 2) gametocyte density, 3) total gametocyte burden, which was calculated as the sum of participant gametocyte densities, and 4) prevalence of infections with ≥10 gametocytes/μL, and there were only two subgroups used for rainy and dry seasonal cohorts. The outcome measurements and analyses were clearly defined and consistent; there was no indication of selection of the reported analysis from among multiple analyses or selection of the cohort or subgroups for analysis and reporting on the basis of the results.

### Effect of intervention.

Baiden et al.^9^ reported on the incidence of malaria in children after the first episode of febrile illness. In the RDT-based intervention arm, there were 914 episodes (incidence rate [IR] = 0.64; 95% CI, 0.49–0.82), and there were 1,011 episodes in the clinical judgement control arm (IR = 0.76; 95% CI, 0.63–0.93). The crude IRR was 1.12 with a 95% CI of 0.81 to 1.54 and a *P* of 0.50. The adjusted IRR was 1.13 (95% CI, 0.82–1.55) with a *P *of 0.47. The incidence of malaria infection for all episodes was also reported and was not statistically significant (intervention, 0.99 per child per year [95% CI, 0.78–1.27]; control, 1.05 per child per year [95% CI, 0.87–1.29; IRR = 1.09 [95% CI, 0.81–1.48]). Based on the results from one study by Baiden et al.,^9^ at 24 months postintervention, TTaT probably results in little to no difference in the incidence of malaria infection (small effect and moderate certainty of evidence).

Adverse events were reported by Halliday et al.^8^ by using a combination of active and passive surveillance in schools up to 28 days postintervention. Active surveillance detected 4.5% (92/2,030) of children reporting one or more AEs within 2 days of receiving treatment, including headache (*n =* 68; 3.3%), stomachache (*n =* 38; 1.9%), dizziness (*n =* 17; 0.8%), vomiting (*n =* 7; 0.3%), and pruritis (*n =* 10; 0.5%). Based on AEs reported by Halliday et al.^8^ using active and passive surveillance, TTaT likely results in little to no difference in AEs in the group targeted by the intervention (moderate certainty of evidence).

**Figure 2.**
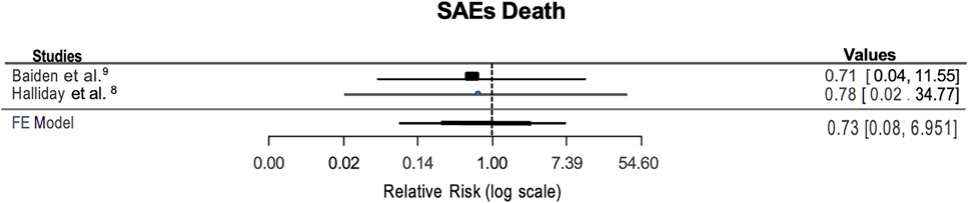
Relative effects of targeted testing and treatment (TTaT) intervention compared with no TTaT intervention on severe adverse events.

Serious AEs were categorized for this review as mortality in study participants. In the report by Halliday et al.,^8^ 11 children died: five in the intervention group and six in the control group during the 24-month follow-up period. The causes of death included yellow fever, heart defect, leukemia, drowning, trauma, pneumonia, and pediatric HIV. None of these deaths in the intervention group occurred within 30 days of the screening and treatment intervention and so were not attributed to study participation. Baiden et al.^9^ also documented deaths during the 24 months of the study. There were 21 deaths in the clinical judgement control arm compared with 15 deaths in the RDT-test based intervention arm. The difference was not considered statistically significant (*P* = 0.31). The statistical synthesis for SAEs in the two studies yielded a proportion of 0.5% in the intervention group compared with 0.7% in the control group and determined that TTaT likely results in little to no difference in AEs and SAEs among the group targeted by the intervention^8^^,^^9^ (RR = 0.73; 95% CI, 0.08–6.95) (moderate certainty of evidence) (Figure 2).

Halliday et al.^8^ reported the prevalence of malaria in the group targeted by the intervention at 12 and 24 months. The adjusted risk ratios (aRR) accounted for school-level clustering, demographics (including age and sex), school mean exam score, and literacy to account for the stratification approach used for concurrent educational intervention. The aRR, however, did not account for *P. falciparum* infection at baseline. The study concluded that TTaT probably does not reduce the prevalence of malaria at 12 months, which was 14.3% in the control arm versus 10.7% in the intervention arm (aRR = 0.71; 95% CI, 0.46–1.11, *P* = 0.131), and also that TTaT probably results in little to no difference in the prevalence of malaria at 24 months, which was 8.5% in the control arm versus 11.8% in the intervention arm (aRR = 1.53; 95% CI, 0.89–2.62, *P* = 0.124) (moderate certainty of evidence).

Cohee et al.^10^ reported on gametocyte prevalence in the rainy season (baseline, 31.1% [27.6–34.5%]; 6 weeks postintervention, 12.8% [9.5–16.1%]) and dry season (baseline, 25.7% [20.9–30.4%]; 6 weeks postintervention, 8.8% (5.4–12.2%)] cohorts. The prevalence of gametocyte-containing infections overall among treated students decreased from 52% (95% CI, 45–58%) at baseline to 11% (95% CI, 7–14%) after 2 weeks, which is a 79% reduction, and gametocyte prevalence remained low after 6 weeks (10% [95% CI, 6–14%]). Among untreated students (RDT negative), the prevalence of gametocyte-containing infections remained relatively unchanged from baseline (9% [95% CI, 5–14%]) to 6 weeks after the intervention (12% [95% CI, 7–17%]). For the combined rainy and dry season cohorts, the prevalence decreased from 28% at baseline to 11% at 6 weeks postintervention. Thus, the study revealed that TTaT resulted in a reduction in prevalence of malaria among the group targeted by the intervention at 6 weeks postintervention; the relative ratio (RR) for crude prevalence of gametocyte-containing infections was 0.43 (95% CI, 0.33–0.55; moderate certainty of evidence). A summary of findings on TTaT compared with no TTaT for reduction of malaria transmission is provided in Table 2.

**Table 2:** GRADE summary of findings: should TTaT vs. no TTaT be used for reducing malaria transmission?

TTaT Compared with No TTaT for Reducing Malaria Transmission						
Outcomes	Anticipated absolute effects* (95% CI)		Relative Effect (95% CI)	Number of Participants (studies)	Certainty of Evidence (GRADE)	Comments
	Risk with No TTaT	Risk with TTaT				
Incidence of malaria infection (community level) Assessed by RDT, microscopy	666/1,000	752/1,000 (546–1,032)	RR 1.13 (0.82–1.55)	3,046 (1 RCT)^†^	⨁⨁⨁◯ Moderate^‡^^§^	TTaT probably results in little to no difference in incidence of malaria infection
AEs (among group targeted by intervention) Assessed by active surveillance	0/1,000	0/1,000 (0–0)	Not estimable	2,030 (1 RCT)‖	⨁⨁⨁◯ Moderate^¶^	TTaT likely results in little to no difference in AEs among the group targeted by intervention
SAEs (among group targeted by intervention) Assessed by mortality	7/1,000	5/1,000 (1–47)	RR 0.73 (0.08–6.95)	8,222 (2 RCTs)^†^‖	⨁⨁⨁◯ Moderate^#^**	TTaT likely results in little to no difference in SAEs in the group targeted by intervention
Prevalence of malaria infection (among group targeted by intervention) Assessed by RDT, microscopy Follow-up: mean, 12 months	143/1,000	102/1,000 (66–159)	RR 0.71 (0.46–1.11)	4,382 (1 RCT)‖	⨁⨁⨁◯ Moderate**	TTaT probably has little to no effect on malaria prevalence in the group targeted by the intervention
Prevalence of malaria infection (among group targeted by intervention) Assessed by: RDT, microscopy Follow-up: mean, 24 months	84/1,000	129/1,000 (75–221)	RR 1.53 (0.89–2.62)	4,140 (1 RCT)‖	⨁⨁⨁◯ Moderate**	TTaT probably results in little to no difference in prevalence in the group targeted by intervention
Prevalence of malaria infection (among group targeted by intervention) Assessed by: RDT, microscopy Follow-up: mean, 6 weeks	255/1,000	110/1,000 (84–140)	RR 0.43 (0.33–0.55)	1,317 (1 observational study)^††^	⨁⨁⨁◯ Moderate**^‡‡^	TTaT reduces the prevalence of malaria in the group targeted by intervention at 6 weeks

AEs = adverse events; GRADE = grading of recommendations, assessment, development, and evaluations; RDT = rapid diagnostic test; SAEs = serious adverse events; TTaT = targeted testing and treatment.

* The risk in the intervention group (and its 95% CI) is based on the assumed risk in the comparison group and the relative effect of the intervention (and its 95% CI).

^†^ See reference ^9^.

^‡^ High risk of bias for domain 5 of RoB2 (Version 2 of the Cochrane risk-of-bias tool for randomized trials) assessment—selection of reported result. Baiden et al.^9^ assessed the incidence of malaria; episodes of malaria accounted for repeat illnesses but did not assess the number of children in the intervention and control arms that had malaria. The incidence was instead categorized by all episodes, i.e., episodes after first fever and repeat malaria, and the prevalence or number of clinical cases was not reported. A multilevel Poisson regression analysis was conducted to calculate the incidence and rate ratios for comparison in study arms, but a generalized model accounting for potential demographics and confounders was not performed to assess the risk of malaria infection in the study arms.

^§^ The incidence among a high-risk population within the community was used as a surrogate for community-level impact.

‖ See reference ^8^.

^¶^ Outcome was not measured in the control arm.

^#^ Imprecision due to wide CIs; crude data used for mortality were unadjusted for additional criteria or potential confounders.

** Absolute effect estimates both appreciable risk and appreciable benefit.

^††^ See reference ^10^.

^‡‡^ Moderate risk of bias in D7 of Robins I (Risk of Bias tool for nonrandomized studies for intervention) - bias in selection of reported results i.e., there were multiple measurements and analyses used within the outcome domain.

### Contextual factors.

Nine articles were assessed for contextual factors. Resource use was assessed based on three studies, by Batwala et al.,^12^ Drake et al.,^13^ and Tawiah et al.^18^ Both Batwala et al.^12^ and Tawiah et al.^18^ conducted cost-effectiveness studies. Tawiah et al.,^18^ linked to the cRCT by Baiden et al.^9^ in Ghana, aimed to evaluate the cost-effectiveness of introducing RDTs in lower-level public health centers where malaria diagnosis was typically presumptive. Researchers found that implementation of test-based malaria case management led to fewer ACT treatments than clinical judgement approach. However, the introduction of RDTs increased the appropriately treated patients by 134 cases, simultaneously increasing the case management cost by $18.60 USD per extra appropriately treated child compared with that of presumptive diagnosis and treatment. From a societal perspective, if costs borne by households were included, the incremental cost effectiveness ratio was further reduced to $11.00 USD per appropriately treated child. In Uganda, the study by Batwala et al.^12^ assessed the cost for three malaria diagnostic strategies in remote primary care centers in accordance with national policies for use of RDTs to target AL treatment to only patients with parasitemia. They found that the use of RDTs increased the cost of diagnosis by $0.67 (an increase of about 108%) in comparison with that by presumptive diagnosis; however, similar to the findings of Tawiah et al.,^18^ the effectiveness of RDT use, if clinicians adhered to test results, provided improved quality of care and cost-effectiveness for overall malaria case management. The use of RDTs was considered the most cost-effective strategy, with an incremental cost-effectiveness ratio of $5.00, compared with microscopy at $9.61 per case correctly diagnosed and treated. Drake et al.,^13^ linked to the cRCT by Halliday et al.,^8^ concluded that IST in schools was a relatively expensive intervention costing $6.61 per child screened. The main costs incurred were for RDTs and salaries for supervised treatment administration. Researchers concluded that to make the intervention more cost effective, cheaper RDTs and unsupervised treatments could reduce costs by up to 47%. Overall, TTaT can be considered cost-effective for targeting children under 5 years of age in comparison with presumptive treatment when using RDTs for diagnosis. However, for school-based implementation, TTaT intervention costs can be substantial and high due to human resource costs and dependent on the selected RDT used.

Feasibility was assessed in one study by Okello et al.,^16^ which assessed the ability to address key challenges during the implementation of the intervention. The researchers highlighted the importance of adequate consent and community engagement processes in school-based research. In addition, they concluded that teachers are critical in school-based health research, so having their support and active involvement is essential for success. For community engagement, the process must be dynamic and responsive to community concerns and needs. Addressing these social aspects during the implementation process and including the community and key stakeholders would contribute to the feasibility of implementing school-based IST.

Values and preferences were reported in two studies, by Okello et al.^15^ and Yan et al.^19^ Okello et al.^15^ found that parents, health workers, and educators all perceived IST in schools to contribute to a reduction in clinical disease and considered malaria control important. However, few participants were aware that the principal aim of the intervention was the reduction of asymptomatic parasitemia rather than the treatment of clinical disease. Yan et al.^19^ assessed the value of malaria testing and reasons why miners may not pursue the recommended course of treatment. Researchers found that miners self-medicate so they can resume mining activities as soon as possible and are willing to pay what is required to address their symptoms. Additionally, miners who sought testing had a belief that correct testing would be beneficial for timely diagnosis and treatment. However, because of side effects of treatment and the financial pressures to return to work, most miners stopped treatment as soon as they felt better. It was evident that services were usually sought when in close proximity, and there was a preference for public service treatment over private services where quality and reliability were sometimes a concern.

Health equity was not addressed by any studies retained for contextual factors. However, in all of the included studies, the demographics review included gender stratifications to ensure balance in the study arms. Results for baseline surveys found no significant differences in infection by gender.

Acceptability was assessed based on five studies, those by Baiden et al.,^11^ Mphwatiwa et al.,^14^ Okello et al. in 2012,^15^ Okello et al. in 2013,^16^ and Taffon et al.^17^ Mphwatiwa et al.^14^ was linked to the cRCT by Halliday et al.^8^ and found that the use of RDTs and ACTs by teachers was an acceptable way of delivering care for malaria to schoolchildren. The benefits of school-based IST included a perception of improved access to malaria treatment for schoolchildren, decreased school absenteeism, and that the program supported broader national health and education policies. Potential barriers to successful implementation disclosed by study participants and implementers included increased teacher workloads, a feeling of inadequate supervision from health workers, lack of incentives, and concerns for sustainability regarding the supply of drugs and commodities.^14^ In one study by Okello et al.,^15^ lack of awareness that IST was intended to treat asymptomatic parasitemia did not appear to impact the acceptability of the intervention. However, some parents expressed concern that their children were given malaria treatment when they were perceived to be healthy and so encouraged their children not to take their medication and instead used the drugs to treat other sick siblings or threw them away. The researchers concluded that while the concept of screening and treatment of malaria is generally acceptable, adherence to treatment given to children with asymptomatic parasitemia may be problematic.^15^ Okello et al.^16^ also found that the intervention in schools was acceptable to teachers and the community, as long as engagement and concerns of community stakeholders were addressed adequately throughout the school-based trial.^16^ In the study by Baiden et al.,^11^ acceptability of RDT-based management of malaria was based on perceptions that confirming a malaria diagnosis before giving an ACT for treatment would improve the likelihood of curative therapy and good prognosis, while management on the basis of clinical judgement was considered speculative and could lead to unfavorable clinical outcomes. Among caregivers for children under 5 years of age, the RDT-based treatment was indicative of improvements in the quality of care and the health system.^11^ In the evaluation by Taffon et al.^17^ the acceptability of the proactive case detection screening activities was related to the perceived simplicity and reliability of the intervention and the perceived efficacy of the drug used for treatment, if positive. The forest-goers and plantation workers that participated in the focus groups indicated that not only was the intervention acceptable but also that those who could not participate hoped to have an opportunity to participate in the future. Lack of participation in voluntary screening and treatment was associated with availability and the need to choose between working in the field or staying in the village to get tested.^17^

### Modeling.

Two studies were retained for modeling. The study by Cohee et al.^10^ conducted a model estimate for the effects of TTaT on community prevalence of malaria infection. Kern et al.^20^ conducted a modeling and simulation analysis using a deterministic compartmentalized model based on the basic parasite cycle of malaria transmission in human and mosquito populations. The deterministic compartmental model allows subjects to move between different states of susceptible, infected, and recovered using estimates for the “infected state” based on a rate determined by mosquito density, the human biting rate, the prevalence of infectiousness in the mosquito population, and the probability of a subject developing a blood-stage infection. Kern et al.^20^ determined through this computer simulation that community screening and treatment targeting vulnerable populations under 5 years of age had the greatest effect when performed in an area with high endemicity (entomological inoculation rate [EIR] > 200).^20^ However, the rate of infection returned to its normal levels in the subsequent year if the intervention was not repeated. The strongest decrease in malaria incidence in the target population was observed with a total of three campaign rounds conducted in close succession, separated by 1 month each for 3 months. When the intervention was implemented 3 months apart, at months 1, 3, and 6 of the dry season, the effectiveness of the intervention was decreased and the subsequent impact on the next malaria season was also reduced. In areas with low disease burden (EIR < 10), the reduction was sustained for over 3 years after a single intervention.^20^

In the simulation reported by Cohee et al.^10^ as a part of their cohort study, an estimated effect of the screen-and-treat intervention could result in at least 6 weeks of reductions in the community prevalence of gametocyte-containing infections (26% in the rainy season and 34% in the dry season) if the intervention was extended to all school attendees in the community. The total gametocyte burden (sum of gametocyte densities) in the community would be reduced by 33% in the rainy season and 25% in the dry season. This estimate was based on the cross-sectional surveys that indicated a comparable gametocyte burden in the community in school-aged children of 47% despite making up approximately 35% of the population.^10^

## DISCUSSION

This systematic review served to collate evidence to further understand how populations at high risk of malaria, and the surrounding populations, could benefit from a TTaT approach to achieve malaria control and elimination. The certainty of evidence for outcomes from estimating the effect of TTaT compared with no TTaT was moderate. The incidence of malaria infection was measured by one study.^9^ Based on a small effect and moderate certainty of evidence, TTaT probably results in little to no difference in incidence of malaria infection at the community level when compared with that resulting from the standard of care using clinical judgement. The increase observed for incidence is expected to be a reflection of improved diagnosis and treatment of febrile patients and not an indication of increased risk of infection; this effect was not statistically significant.^9^ Adverse events and SAEs both had a moderate certainty of evidence based on two studies, by Baiden et al.^9^ and Halliday et al.,^8^ which determined that TTaT likely results in little to no difference in AEs and SAEs among the group targeted by the intervention.^8^^,^^9^ Evidence for the prevalence of malaria infection was assessed based on results from one study by Halliday et al.^8^ at 12- and 24-month follow-up periods postintervention. These two time points revealed inconsistent results, neither of which had a statistically significant relative effect. The study results suggested that at 12 months postintervention, TTaT probably does not reduce the prevalence of malaria, and at 24 months, TTaT probably results in little to no difference in the prevalence of malaria.^8^ Furthermore, a modeling simulation by Kern et al.^20^ found that the rate of infection would return to normal levels in the subsequent year if the intervention was not repeated in an area where malaria endemicity was moderate to high.^20^ In the region in Kenya where the Halliday et al.^8^ study was conducted, the EIR was estimated at 231 to 269 infective bites per person per year, and in the Kern et al.^20^ simulation, the EIR was > 200 infective bites per person per year. Although the modeling simulation used community screening and treatment, Kern et al. did assess the impact based on a targeted high-risk population of children under 5 year old.^20^ The prevalence of malaria infection was also assessed at 6 weeks postintervention in one nonrandomized study by Cohee et al.^10^ The study had a moderate certainty of evidence and indicated that TTaT reduced malaria prevalence among the group targeted by the intervention. In their simulated results using similar time frames, Cohee et al.^10^ also estimated that the community impact of TTaT would be similar to what was exhibited in the study population. Model estimates based on community data projected a 26% decline in prevalence in the rainy season and a 34% decline in the dry season.

At the time of the initial bibliographical search (March 2021) and update (April 2022), four clinical trials^21^^–^^24^ that met the inclusion criteria were still ongoing. Of these four trials, one cRCT, open-label, superiority trial conducted in 757 infants in 21 clusters of village health posts and followed from 6 weeks of age until 12 months between 2014 and 2017 in Papua, Indonesia, was published in June 2022.^25^ The trial aimed to determine the effects of intermittent screening and treatment of infants (ISTi) with malaria on morbidity in a high-malaria-transmission area. During the ISTi (with or without malaria symptoms), infants participating in the intervention group were screened using an RDT and microscopy at 2, 3, 4, and 9 months and were treated with dihydroartemisinin-piperaquine (DHP) for 3 days, whereas infants in the control group were screened only if they had clinical symptoms during the study period and were treated, if positive, with DHP. Additionally, infants in both the intervention and control groups were screened with RDTs and microscopy at 6 months and with RDTs, microscopy, and quantitative polymerase chain reaction (qPCR) at 12 months. The two main outcomes of the study were the incidence of clinical malaria in the first 12 months of life and the prevalence of malaria infection in the group targeted by the intervention at 12 months of age. The study revealed that there was no significant difference in incidence of clinical malaria, i.e., an adjusted incidence rate ratio (aIRR) of 1.77 (95% CI, 0.62–5.01, *P* = 0.280). The prevalence at 12 months was 15% in the control arm and 13% in the intervention arm with an adjusted prevalence ratio (aPR) of 0.92 (95% CI, 0.70–1.21, *P* = 0.55), and no significant difference was found between the intervention and control arms.

In studies assessing contextual factors, three reports indicated that TTaT using RDT-based testing could be considered cost-effective for targeting children under 5 years of age in comparison with presumptive treatment.^12^^,^^13^^,^^18^ However, school-based TTaT implementation can be costly because of human resource costs and depending on the selected RDT used.^13^ Okello et al. considered the implementation of school-based IST feasible as long as community engagement and key stakeholders were incorporated into the process.^16^ Okello et al.^15^ and Yan et al.^19^ both determined that TTaT was valued as an intervention aimed to decrease malaria incidence and preferred the use of testing for diagnosis prior to treatment.^15^^,^^19^ However, in the study by Okello et al.,^15^ it was noted that when asymptomatic parasitemia was targeted, it was often not understood since children without symptoms were perceived as healthy, causing parents some reluctance to give children the medication.^15^ Acceptability was assessed based on five studies, all of them concluding that TTaT is an acceptable intervention in a variety of settings due to the simplicity of the intervention, perceived indication for improved quality of care, perceived efficacy of test-based diagnosis used to inform treatment, and subsequently prognosis.^11^^,^^14^^–^^17^ However, when the intervention to target and treat children with asymptomatic parasitemia is used, there may be issues with compliance since there is no clinical disease evident to caregivers.^11^^,^^14^^–^^17^

Future iterations of the review should consider asymptomatic parasitemia and gametocyte carriage as the main outcomes. While individuals with these characteristics may not be considered high risk due to disease severity or outcome, they may represent the population that poses a significant contribution to transmission in moderate- and low-transmission settings and serve as an important reservoir of infection, as indicated in the Cohee et al.^10^ study in southern Malawi in schoolchildren. In addition, submicroscopic parasite carriage is considered common in low-endemicity settings and in chronic infections.^15^ Targeting this population can improve surveillance efforts for understanding the epidemiology of malaria infection within a population and help to estimate the true burden of infection in a community; therefore, TTaT could be used as a surveillance strategy. Moreover, given that these individuals can still facilitate transmission to mosquitoes but can often be missed by RDTs, they can be considered significant in perpetuating the transmission cycle, as well as a neglected group for treatment if clinical care is not perceived as needed or sought after because of the lack of clinical disease. In the study by Cohee et al.,^10^ 16% (unweighted, 38/180) of all students with gametocyte-containing infections and 9% (unweighted, 4/32) with infections containing ≥10 gametocytes/µL were RDT negative and so were not treated as a part of the school-based IST intervention. Rapid diagnostic tests were less likely to detect gametocyte-containing infections at lower densities.^10^ The difficulty is that in order to identify asymptomatic infections, a TTaT approach will work only if the targeted high-risk population is also the population that harbors the major reservoir of asymptomatic infections. Some studies have carried out community-wide mass screen-and-treat interventions^26^^,^^27^ and so were not eligible for inclusion in this review; however, an aim in those studies also included targeting a high-risk population, such as children under 5 years, schoolchildren, and asymptomatic carriers, but used a mass screening approach to assess the impact on vulnerable groups similar to the modeling simulation included by Kern et al.^20^

An accurate diagnosis is the cornerstone for initiating timely malaria treatment and controlling transmission in countries where the disease is endemic. Although RDTs are cheap, readily available, and easy to use and interpret, the performance of RDTs depends on various factors, including parasite density, transmission setting, and individuals’ parameters such as age and immunity.^28^ Likewise, despite the many disadvantages, including a lack of sensitivity and specificity, microscopy is still considered a rapid and cost-effective (“gold standard”) test for diagnosing malaria.^29^^,^^30^ Despite these limitations, the WHO has recommended RDTs and/or microscopy for parasitological confirmation before the initiation of treatment on the basis of the benefits these tests offer against the risk associated with use.^29^ All the TTaT studies included in this review used RDTs and/or microscopy to screen the participants and mentioned the limitations of these diagnostic tools in detecting submicroscopic infections,^29^ as highlighted by Cohee et al.,^10^ Baiden et al.,^9^ Halliday et al.,^8^ and Poespoprodjo et al.^25^ This is why several studies are now using molecular techniques like PCR, which offers an alternative to microscopy with superior sensitivity in detecting the malaria parasite DNA. This molecular technique is not only expensive but also comes with some challenges, particularly for resource-limited settings, such as required trained personnel, quality control, and maintenance of equipment.^29^ Hence, countries reaching elimination with submicroscopic infections^29^ or conducting prevalence studies should consider PCR as a referral option in research laboratories or other field-friendly molecular tools, such as the loop-mediated isothermal amplification.^31^

Studies using innovative strategies for voluntary test and treat, such as that by Taffon et al.^17^ describing forest-goers and plantation workers in Cambodia, or for self-test and treat, as described in gold miners in French Guiana by Douine et al.,^32^ have increasingly aimed to address access to screening and treatment of high-risk populations known to be key reservoirs of infection in their respective regions.^17^^,^^32^ The focus is now shifting towards school-based interventions because they target both a high-risk group and, in some settings, an important reservoir of infection.^8^^,^^10^^,^^33^ However, it is important to note that despite potentially large impacts on both the targeted population and community transmission, studies included in this review did not identify significant long-term effects of the intervention in the study populations.^8^^,^^10^^,^^20^ In a systematic review of school-based preventative treatment of malaria that assessed the estimated effect of varied school-based programs, including intermittent preventive treatment, chemoprevention, and IST, there was a 72% reduction in the prevalence of *P. falciparum* infection in the study-level meta-analysis and treatment was considered effective across all transmission settings.^34^ The analyses included 11 studies and showed strong evidence of heterogeneity across studies, which could not be explained by follow-up time postintervention among other intervention factors.^34^ Short-term impacts at 6 weeks follow-up were evident in the study by Cohee et al.,^10^ whereas Halliday et al.^8^ found no statistically significant benefit at 12- or 24-month follow-up periods postintervention.^8^ This was perhaps due to differences in the setting of the study; Halliday et al.^8^ found considerable heterogeneity in transmission in the area and assumed that the possibility for rapid reinfection was high.^8^

The two other factors that influence the effect of TTaT are the magnitude of intervention coverage and the frequency of TTaT in a high-risk population. Various factors like reinfection rate, transmission pattern, and duration of prophylaxis provided by the drug of choice should be considered to define the frequency of intervention. Coverage of such programs can be increased by integrating malaria screening and treatment into school health packages or basic health packages offered to workers deployed in high-risk conditions. Hence, this would improve the access to health care in this high-risk population and promote sustainability in high-burden areas.^10^

This systematic review aimed to assess the relative effects of TTaT in high-risk populations based on demographics, occupation, and other exposure characteristics; however, the PICO question did not account for targeting the important reservoirs of infection, which are key in assessing the impact of a targeted strategy. As previously stated, once transmission has declined to the point that it can be considered focal and high-risk populations are identifiable, targeted strategies may be efficacious in reducing transmission even further by targeting a group that represents a large proportion of the infectious reservoir. Moreover, an important aspect of elimination strategies includes targeting those specific high-risk groups that may not be captured through routine prevention and treatment services, which may include asymptomatic carriers of infection. Therefore, after examining the results of this systematic review and deliberations by the WHO Guideline Development Group (GDG), it was concluded that TTaT could be beneficial only in specific circumstances: 1) in small groups of people if the parasite reservoir is limited and easily detectable; 2) if chemoprevention is not acceptable to the population; and 3) if prevention of *P. vivax* with safe and effective radical cure is feasible only for those with confirmed infections.^3^ Although all the studies that met the eligibility criteria and were included in this review were related to *P. falciparum*, the WHO GDG group extrapolated the findings to *P. vivax*. Future iterations of this review should ensure consideration for populations proven to host the vast majority of the reservoir of infection in lower-transmission settings to determine the effectiveness of the intervention.

## Supplemental Materials

10.4269/ajtmh.23-0097 Supplemental Materials
