# Supplementary material for: Targeted Testing and Treatment To Reduce Human Malaria Transmission in High-Risk Populations: A Systematic Review
Source: Am J Trop Med Hyg. 2024 Mar 12;110(4 Suppl):54–64. doi: 10.4269/ajtmh.23-0097 (PMC10993793; doi:10.4269/ajtmh.23-0097)

Supplemental Data

Targeted test and treat for Reduction of Human Malaria Transmission in High-Risk Populations: A Systematic Review

Beena Bhamani,[1*†] Elisabet Martí Coma-Cros,[1*] Maria Tusell[1], Vita Mithi,[2,3,4] Elisa Serra-Casas,[1] Nana Aba Williams,[1] Kim A. Lindblade,[5] Koya C. Allen[1]

[1]Barcelona Institute for Global Health (ISGlobal), Hospital Clínic – University of Barcelona, Barcelona, Spain; [2]Armref Data for Action in Public Health Research Consultancy, Mzuzu, Malawi; [3]Society for Research on Nicotine and Tobacco-Genetics and Omics Network, Madison, USA; [4]Leaders of Africa Institute, Baltimore, USA; [5]Global Malaria Programme, World Health Organization, Geneva, Switzerland

[*] These authors contributed equally.

[†] Address correspondence to Beena Bhamani, Barcelona Institute for Global Health (ISGlobal), Hospital Clínic – University of Barcelona, Carrer Rosselló 171, Entl. 2a, Barcelona 08036, Spain. E-mail: beena.bhamani@isglobal.org

Supplementary Table 1: Search Strategy

	**Search term**
	*malaria/
	exp malaria, falciparum/ or exp malaria, vivax/
	malaria ovale.mp. or Plasmodium ovale/
	plasmodium malariae.mp. or Plasmodium malariae/
	1 or 2 or 3 or 4
	Antimalarials/
	Disease Eradication/ or elimination.tw
	(tailored adj2 (intervention* or treatment* or strateg* or administration)).tw
	(relapse adj2 prevention).mp
	Presumptive adj2 (treatment or therapy).tw
	focal adj2 (drug administration).tw or “focal MDA” or “focal testing and treatment”.tw or FTAT.tw
	targeted adj2 (intervention* or treatment or strateg* or administration).tw
	6 or 7 or 8 or 9 or 10 or 11 or 12
	(“Forest-goers “ or Forests/ or Vulnerable or Worksites or Farm* OR plantation* or mining or miner* or Miners/ or laborers or cultivators or military or “armed forces” or Military Personnel/ or “peace-keepers” or agricultural)
	“high risk population*”.mp. or Risk Factors/
	“special populations”.tw
	“high exposure”.tw or “highly exposed”.tw or “high transmission”.tw or hotspot*.tw
	14 or 15 or 16 or 17
	Diagnostic Techniques and Procedures/
	(screening or screened or diagnosed or diagnostics or test*).tw
	Point-of-Care Testing/
	“rapid diagnostic test*” or RDT.tw
	“parasitological adj2 diagnosis”.tw
	“Parasitologic adj test*”. tw or “symptom screening”.tw
	“case detection” or PACD or ACD
	19 or 20 or 21 or 22 or 23 or 24 or 25
	5 and 13 and 18 and 26

Supplementary Table 2. List of studies excluded after full review and primary reasons for exclusion

Study	Reference	Reason for exclusion
**Amboko (2022)**	1	Population – all outpatient population were targeted
**Baiden (2013)**	2	Cross-referenced or linked article; Not Retrieved - conference proceeding/abstract only
**Baiden (2013)**	3	Cross-referenced or linked article; Not Retrieved - conference proceeding/abstract only
**Bousema (2013)**	4	Population - geographic hotspots not targeted to high risk populations; Protocol
**Brooker (2010)**	5	Protocol -linked to Halliday 2014
**Hast (2018)**	6	Not retrieved - conference proceedings/abstract only
**Kachur (2016)**	7	Study Focus Area - commentary on policy
**Krisher (2016)**	8	Study Focus Area/Study Design - case study on an elimination program approach not specifically TTaT
**Kunkel (2021)**	9	Intervention -passive case and reactive case detection in intervention 'arm'; comparator was observational baseline study in same/comparable population.
**Lausatianragit (2018)**	10	Not retrieved - conference proceedings/abstract only
**Lon (2015)**	11	Not retrieved - conference proceedings/abstract only
**Lover (2018)**	12	Not retrieved - conference proceedings/abstract only
**Lover (2019)**	13	Protocol * if study results are available -study can be included for both TTaT and MTaT
**Nct (2018)**	14	not retrieved - trial registration / intervention & comparison - 2nd study arm weekly active case detection for CCM
**Ndyomugyenyi (2012)**	15	Not retrieved - conference proceedings/abstract only
**Nguon (2018)**	16	Not retrieved - conference proceedings/abstract only / Study design - uncontrolled cross-sectional survey, doesn't seem to be an intervention study
**Rossi (2018)**	17	Intervention - TTaT was combined with RCD / Linked to Taffon Pierluigi (2018) which is included for contextual factors
**Wang (2019)**	18	Protocol; Study focus area - programmatic evaluation of policy
**Tsarafihavy (2018)**	19	Not retrieved - conference abstract
**Ahmed (2019)**	20	Population - Pregnant women; Comparison - compared to a policy recommendation for IPTp as the 'standard of care'
**Douine (2018)**	21	Pilot paper on protocol and intervention description

**Halliday (2012)**	22	Cross-referenced article - linked to Halliday 2014; Brooker 2010
**Hoyer (2012)**	23	Intervention - geographic high-risk locations with mass testing for id of asymptomatic infections; study design - cross sectional survey
**Malaria Consortium (2019)**	24	Not retrieved-report from malaria consortium/protocol/brief
**Poespoprodjo (2018)**	25	Not retrieved - clinical trial registration
**Sutanto (2021)**	26	Not retrieved - clinical trial registration
**Sondo (2021)**	27	Not retrieved - clinical trial registration
**Thangadurai (2020)**	28	Not retrieved - conference abstract
**Tseroni (2020)**	29	Intervention - included reactive case detection in epi investigation of positive reports
**Adinan (2015)**	30	Exclude for contextual factors; Study Focus Area - descriptive secondary analysis of surveillance data
**Babigumira (2017)**	31	Exclude for contextual factors; Study Focus Area - textbook review of cost effectiveness; not a study
**Bennett (2019)**	32	Not-retrieved- abstract/conference proceeding/ cross-referenced article
**Canavati (2019)**	33	Exclude for contextual factors -Did not include CF informaton - included participant interviews from self-reported malaria suspicion and health-seeking to local clinic
**Castellani (2016)**	34	Exclude for contextual factors - based on a CHW intervention not TTaT
**Chritz (2017)**	35	Not retrieved - conference proceedings/abstract only
**Dantzer (2018)**	36	Not retrieved - conference proceedings/abstract only / cross-referenced study abstract
**Dantzer (2019)**	37	Not retrieved - conference proceedings/abstract only/ cross-referenced study abstract
**Fançony (2021)**	38	Intervention - test and treat was implemented as the control for effectiveness trial of other interventions
**Hustedt (2018)**	39	Not retrieved - conference proceedings/abstract only
**Lopes (2020)**	40	Exclude for contextual factors - assessment of knowledge in HCWs
**Nct (2018)**	41	Not retrieved - trial registration
**Nct (2018)**	42	Not retrieved; Exclude for contextual factors
**Olapeju (2020)**	43	Exclude for contextual factors - knowledge, attitudes and practices survey
**Pulford (2016)**	44	Exclude for contextual factors - fever case management evaluation study

**Saad (2018)**	45	Not retrieved - conference abstract; Exclude for contextual factors; Cross-referenced article -linked to Rossi(2018) and Pierluigi (2018)
**Staff (2017)**	46	Cross-referenced to Tawiah(2016) - table correction was published
**Wijesinghe (2011)**	47	Exclude for contextual factors - study focused on acceptability and perceptions of malaria RDTs and ACTs not the intervention/approach of TTaT
**Zurovac (2014)**	48	Exclude for contextual factors - study focuses on fever case management in health facilities
**Eckhoff (2011)**	49	Exclude for modeling; Not retrieved - conference proceedings/abstract only
**Gerardin (2017)**	50	Exclude for modeling; Not retrieved - conference proceedings/abstract only
**Getachew (2016)**	51	Exclude for contextual factors; focused on preferred interventions by migrant workers and who referred participants to a health post; not on TTaT
**Hill (2016)**	52	Exclude for contextual factors; population includes pregnant women and IPTp
**Hill (2018)**	53	Exclude for contextual factors; population includes pregnant women and IPTp
**Hoyt (2018)**	54	Exclude for contextual factors; population includes pregnant women and IPTp

1. Amboko B, Stepniewska K, Machini B, Bejon P, Snow RW, Zurovac D., 2022. Factors influencing health workers' compliance with outpatient malaria 'test and treat' guidelines during the plateauing performance phase in Kenya, 2014–2016. *Malar J 21*: 1–11

2. Baiden F, Bruce J, Webster J, Tivura M, Delmini R, Amenga-Etego S, Owusu-Agyei S, Chandramohan D., 2013. Restricting artemisinin-based combination therapy to test positive malaria in a high-transmission setting in Ghana - a cluster-randomised trial. *Tropical medicine & international health*, 74–74

3. Baiden F, Bruce J, Webster J, Tivura M, Delmini R, Amenga-Etego S, Owusu-Agyei S, Chandramohan D., 2013. Effects of restricting artemisinin-based combination therapy to test positive malaria in a high-transmission setting in Ghana - A cluster-randomized trial. *AJTMH*

4. Bousema T, Stevenson J, Baidjoe A, Stresman G, Griffin JT, Kleinschmidt I, Remarque EJ, Vulule J, Bayoh N, Laserson K, Desai M, Sauerwein R, Drakeley C, Cox J., 2013. The impact of hotspot-targeted interventions on malaria transmission: Study protocol for a cluster-randomized controlled trial. *Trials 14*: 1–12

5. Brooker S, Okello G, Njagi K, Dubeck MM, Halliday KE, Inyega H, Jukes MCH., 2010. Improving educational achievement and anaemia of school children: design of a cluster randomised trial of school-based malaria prevention and enhanced literacy instruction in Kenya. *Trials 11*: 1–14

6. Hast M, Searle K, Lubinda J, Hamapumbu H, Lupiya J, Mamini E, Shields T, Mharakurwa S, Gwanzura L, Munyati S, Stevenson J., 2018. The use of GPS data loggers to describe spatiotemporal movement patterns and the implications for malaria control in three epidemiologic settings in Southern Africa. *American Journal of Tropical Medicine and Hygiene*. AMER SOC TROP MED & HYGIENE, 98–99

7. Kachur SP., 2016. ‘Beyond “test and treat” – malaria diagnosis for improved pediatric fever management in sub-Saharan Africa’ by Emily White Johansson. *https://doi.org/103402/gha.v934416 9*: 34416

8. Krisher LK, Krisher J, Ambuludi M, Arichabala A, Beltrán-Ayala E, Navarrete P, Ordoñez T, Polhemus ME, Quintana F, Rochford R, Silva M, Bazo J, Stewart-Ibarra AM., 2016. Successful malaria elimination in the Ecuador-Peru border region: epidemiology and lessons learned. *Malar J 15*: 1–15

9. Kunkel A, Nguon C, Iv S, Chhim S, Peov D, Kong P, Kim S, Im S, Debackere M, Khim N, Popovici J, Srun S, Vantaux A, Guintran JO, Witkowski B, et al., 2021. Choosing interventions to eliminate forest malaria: preliminary results of two operational research studies inside Cambodian forests. *Malar J 20*: 1–13

10. Lausatianragit K, Sudathip P, Chaitaveep N, Nonkaew P, Roh M, Feldman M, Raseebut C, Tabprasit S, Sriwichai S, Arsanok M, Kuntawunginn W., 2018. Coordinated Thailand ministry of public health and royal Thai army-us army civilian-military response to a malaria outbreak in Northeast Thailand. *Americal Journal of Tropical Medicine and Hygiene*. AMER SOC TROP MED & HYGIENE, 456–456

11. Lon C, Manning J, Somethy S, Chann S, Sriwichai S, Rekol H, Dysoley L, Sinoun M, Satharath P, Saunders D., 2015. Defining effective, appropriate, implementable strategies for malaria elimination in military forces in Cambodia as a model for mobile populations. *American Journal of Tropical Medicine and Hygiene*. AMER SOC TROP MED & HYGIENE, 262–262

12. Lover A, Dantzer E, Hongvanthong B, Hocini S, Phongluxa K, Rerolle F, Phetsouvanh R, Estera R, Smith J, Yukich J, Hwang J., 2018. A community-randomized trial assessing the effectiveness of targeted active malaria case detection among high-risk populations in southern Lao PDR: Study design and baseline survey results. *American Journal of Tropical Medicine and Hygiene*. AMER SOC TROP MED & HYGIENE, 104–104

13. Lover AA, Dantzer E, Hocini S, Estera R, Rerolle F, Smith JL, Hwang J, Gosling R, Yukich J, Greenhouse B, Jacobson J, Phetsouvanh R, Hongvanthong B, Bennett A., 2019. Study protocol for a cluster-randomized split-plot design trial to assess the effectiveness of targeted active malaria case detection among high-risk populations in Southern Lao PDR (the AcME-Lao study). *Gates Open Res 3*: 1730

14. Anon. P. Falciparum Infection Dynamics and Transmission to Inform Elimination (INDIE-1a) - Full Text View - ClinicalTrials.gov. Available at: https://clinicaltrials.gov/ct2/show/NCT03705624. Accessed

15. Ndyomugyenyi R, Hansen KS, Lal S, Chandler C, Mbonye AK, Magnussen P, Clarke SE., 2012. Introducing rapid diagnostic tests into community-based management of malaria: Evidence from a cluster-randomized trial in two areas of high and low transmission in Uganda. *AJTMH*

16. Nguon S et al., 2018. Accessing Hard-to-Reach Populations: Forest interventions increase case detection in Pursat Province, Cambodia. Available at: https://www.abstractsonline.com/pp8/#!/4692/presentation/19191. Accessed. 2018

17. Rossi G, Vernaeve L, van den Bergh R, Nguon C, Debackere M, Abello Peiri C, Van V, Khim N, Kim S, Eam R, Ken M, Khean C, de Smet M, Menard D, Kindermans JM., 2018. Closing in on the Reservoir: Proactive Case Detection in High-Risk Groups as a Strategy to Detect Plasmodium falciparum Asymptomatic Carriers in Cambodia. *Clin Infect Dis 66*: 1610–1617

18. Wang D, Chaki P, Mlacha Y, Gavana T, Michael MG, Khatibu R, Feng J, Zhou Z bin, Lin KM, Xia S, Yan H, Ishengoma D, Rumisha S, Mkude S, Mandike R, et al., 2019. Application of community-based and integrated strategy to reduce malaria disease burden in southern Tanzania: the study protocol of China-UK-Tanzania pilot project on malaria control. *Infect Dis Poverty 8*

19. Tsarafihavy A, Razanakatovo T, Yanulis J, Thurow A, Hall T, Kapesa L, Razafindrakoto J., 2018. From Presumed to Confirmation Diagnosis:Improving testing and treatment of malaria in children under five in rural communities in Madagascar. Available at: https://www.abstractsonline.com/pp8/#!/4692/presentation/14698. Accessed. 2018

20. Ahmed R, Poespoprodjo JR, Syafruddin D, Khairallah C, Pace C, Lukito T, Maratina SS, Asih PBS, Santana-Morales MA, Adams ER, Unwin VT, Williams CT, Chen T, Smedley J, Wang D, et al., 2019. Efficacy and safety of intermittent preventive treatment and intermittent screening and treatment versus single screening and treatment with dihydroartemisinin–piperaquine for the control of malaria in pregnancy in Indonesia: a cluster-randomised, open-label, superiority trial. *Lancet Infect Dis 19*: 973–987

21. Douine M, Sanna A, Galindo M, Musset L, Pommier De Santi V, Marchesini P, Magalhaes ED, Suarez-Mutis M, Hiwat H, Nacher M, Vreden S, Garancher L., 2018. Malakit: an innovative pilot project to self-diagnose and self-treat malaria among illegal gold miners in the Guiana Shield. *Malar J 17*

22. Halliday KE, Karanja P, Turner EL, Okello G, Njagi K, Dubeck MM, Allen E, Jukes MCH, Brooker SJ., 2012. Plasmodium falciparum, anaemia and cognitive and educational performance among school children in an area of moderate malaria transmission: baseline results of a cluster randomized trial on the coast of Kenya. *Trop Med Int Health 17*: 532–549

23. Hoyer S, Nguon S, Kim S, Habib N, Khim N, Sum S, Christophel EM, Bjorge S, Thomson A, Kheng S, Chea N, Yok S, Top S, Ros S, Sophal U, et al., 2012. Focused Screening and Treatment (FSAT): A PCR-Based Strategy to Detect Malaria Parasite Carriers and Contain Drug Resistant P. falciparum, Pailin, Cambodia. *PLoS One 7*: e45797

24. Anon. Malaria Consortium - Countering the spread of drug-resistant malaria in northern Cambodia. Available at: https://www.malariaconsortium.org/resources/publications/1308/countering-the-spread-of-drug-resistant-malaria-in-northern-cambodia. Accessed

25. Anon. Malaria in Early Life Study - Full Text View - ClinicalTrials.gov. Available at: https://clinicaltrials.gov/ct2/show/NCT02001428. Accessed

26. Anon. Serological Screen and Treat Trial for Plasmodium Vivax - Full Text View - ClinicalTrials.gov. Available at: https://clinicaltrials.gov/ct2/show/NCT04223674. Accessed

27. Anon. Boosting the Impact of SMC Through Simultaneous Screening and Treatment of Roommates - Full Text View - ClinicalTrials.gov. Available at: https://clinicaltrials.gov/ct2/show/NCT04816461. Accessed

28. Thangadurai S, et al., 2020. Forest malaria interventions have made remarkable progress in drastically reducing Plasmodium falciparummalaria in Pursat Province, Cambodia. *AJTMH*

29. Tseroni M, Georgitsou M, Baka A, Pinaka O, Pervanidou D, Tsironi M, Bleta P, Charvalakou M, Psinaki I, Dionysopoulou M, Legaki A, Vakali A, Patsoula E, Vassalou E, Bellou S, et al., 2020. The Importance of an Active Case Detection (ACD) Programme for Malaria among Migrants from Malaria

Endemic Countries: The Greek Experience in a Receptive and Vulnerable Area. *Int J Environ Res Public Health 17*

30. Adinan J, Damian DJ, Msuya SE., 2015. Factors Associated with Testing and Prompt Use of Recommended Antimalarials following Malaria Diagnosis: A Secondary Analysis of 2011-12 Tanzania HIV and Malaria Indicator Survey Data. *PLoS One 10*

31. Babigumira JB, Gelband H, Garrison LP, Jr., 2017. Cost-Effectiveness of Strategies for the Diagnosis and Treatment of Febrile Illness in Children. *Disease Control Priorities, Third Edition (Volume 6): Major Infectious Diseases*: 385–400

32. Bennett A, Dantzer E, Hongvanthong B, Rerolle F, Hocini S, Smith J, Hwang J, Gosling R, Yukich J, Greenhouse B, Phetsouvanh R., 2019. Targeted Surveillance for Forest-based malaria transmission: Results of a cluster randomized controlled trial in southern Lao PDR. *American Journal of Tropical Medicine and Hygiene*. AMER SOC TROP MED & HYGIENE, 216–216

33. Canavati SE, Kelly GC, Quintero CE, Vo TH, Tran LK, Ohrt C, Ngo TD, Tran DT, Martin NJ., 2019. Risk factor assessment for clinical malaria among forest-goers in a pre-elimination setting in Phu Yen Province, Vietnam. *Malar J 18*: 1–10

34. Castellani J, Nsungwa-Sabiiti J, Mihaylova B, Ajayi IO, Siribié M, Afonne C, Balyeku A, Sermé L, Sanou AK, Sombié BS, Tiono AB, Sirima SB, Kabarungi V, Falade CO, Kyaligonza J, et al., 2016. Impact of Improving Community-Based Access to Malaria Diagnosis and Treatment on Household Costs. *Clin Infect Dis 63*: S256

35. Chritz S, Yeshiwondim AK, Bansil P, Workie WM, Agma AA, Zeleke MT, Guesses GS, Serda BA, Tesfay BH, Kidanemariam TG, Salisbury N, Earle D, Steketee RW, Guinovart C, Getachew A. Malaria control in migrant laborers working in agricultural farms in Metema Region, Ethiopia: Current practices, feasibility, and acceptability of new malaria interventions

36. Dantzer E, Lover A, Hongvanthong B, Hocini S, Phongluxa K, Phetsouvanh R, Bennett A., 2018. Feasibility and acceptability of a peer navigator led malaria focal test and treat intervention targeting high-risk populations in Southern Lao PDR. *American Journal of Tropical Medicine and Hygiene*. AMER SOC TROP MED & HYGIENE, 354–354

37. Dantzer E, Lover AA, Hongvanthong B, Phongluxa K, Rerolle F, Hocini S, Phetsouvanh R, Bennett A., 2019. Feasibility and acceptability of a peer navigator led malaria focal test and treat intervention targeting high-risk populations in southern Lao PDR. *American Journal of Tropical Medicine and Hygiene*. AMER SOC TROP MED & HYGIENE, 524–525

38. Fançony C, Soares Â, Lavinha J, Barros H, Brito M., 2021. Effectiveness of Nutrition and WASH/malaria educational community-based interventions in reducing anemia in children from Angola. *Scientific Reports 2021 11:1 11*: 1–12

39. Anon. Malaria Consortium - Expanding the health system into the forest: Analysis of and response to the challenge of providing malaria services inside forest areas within the Greater Mekong Subregion in the context of malaria elimination. Available at: https://www.malariaconsortium.org/resources/publications/1182/expanding-the-health-system-into-the-forest-analysis-of-and-response-to-the-challenge-of-providing-malaria-services-inside-forest-areas-within-the-greater-mekong-subregion-in-the-context-of-malaria-elimination. Accessed

40. Lopes SC, Mugizi R, Pires JE, David F, Martins J, Dimbu PR, Fortes F, Rosário J, Allan R., 2020. Malaria Test, Treat and Track policy implementation in Angola: A retrospective study to assess the progress achieved after 4 years of programme implementation. *Malar J 19*: 1–12

41. Anon. Routine Antenatal Care Versus Screening and Treatment of Malaria in Pregnancy in Rwanda - Full Text View - ClinicalTrials.gov. Available at: https://clinicaltrials.gov/ct2/show/NCT03508349. Accessed

42. Anon. Targeted Active Case Detection Among High Risk Populations in Southern Lao Peoples Democratic Republic - No Study Results Posted - ClinicalTrials.gov. Available at: https://clinicaltrials.gov/ct2/show/results/NCT03783299. Accessed

43. Olapeju B, Adams C, Hunter G, Wilson S, Simpson J, Mitchum L, Davis T, Orkis J, Cox H, Trotman N, Imhoff H, Storey D., 2020. Malaria prevention and care seeking among gold miners in Guyana. *PLoS One 15*: e0244454

44. Pulford J, Smith I, Mueller I, Siba PM, Hetzel MW., 2016. Health Worker Compliance with a 'Test And Treat' Malaria Case Management Protocol in Papua New Guinea. *PLoS One 11*: e0158780

45. Saad NJ, et al., 2018. Pro-active case detection in an area of artemisinin resistance: identifying asymptomatic carriers and accelerating elimination efforts. *AJTMH*

46. Anon. Correction: Cost-Effectiveness Analysis of Test-Based versus Presumptive Treatment of Uncomplicated Malaria in Children under Five Years in an Area of High Transmission in Central Ghana - PubMed. Available at: https://pubmed.ncbi.nlm.nih.gov/28107534/. Accessed

47. Wijesinghe RS, Atkinson JAM, Bobogare A, Wini L, Whittaker M., 2011. Exploring provider and community responses to the new malaria diagnostic and treatment regime in Solomon Islands. *Malar J 10*: 1–12

48. Zurovac D, Githinji S, Memusi D, Kigen S, Machini B, Muturi A, Otieno G, Snow RW, Nyandigisi A., 2014. Major Improvements in the Quality of Malaria Case-Management under the "Test and Treat" Policy in Kenya. *PLoS One 9*: e92782

49. Eckhoff P., 2011. Modeling for malaria elimination in a variety of transmission settings. Available at: https://www.abstractsonline.com/Plan/ViewAbstract.aspx?sKey=e085170f-1e5f-491f-99e6-cf35f6c71b8a&cKey=480e41d3-de87-43bb-9e31-7fc88af3536e&mKey=%7b6BADBAB5-8298-4B1A-B5C2-0EEFA5120BB0%7d. Accessed. 2011

50. Gerardin J, Bever CA, Bridenbecker D, Eisele TP, Miller JM, Eckhoff PA, Wenger EA., 2017. Preventing reestablishment of malaria in recently eliminated areas: A modeling study of reactive case detection and adaptive response. *American Journal of Tropical Medicine and Hygiene*. AMER SOC TROP MED & HYGIENE, 593–594

51. Anon. Migrant Labor Report Ethiopia 2016: Assessing movement patterns and malaria-associated factors among agricultural migrant workers and the feasibility of implementing new targeted anti-malaria interventions in Amhara Region, Ethiopia | PATH. Available at: https://www.path.org/resources/migrant-labor-report-ethiopia-2016-assessing-movement-patterns-and-malaria-associated-factors-among-agricultural-migrant-workers-and-feasibility-implementing-new-targeted-anti-malaria-interventions-amhara-region-ethiopia/. Accessed

52. Hill J, Hoyt J, Achieng F, Ouma P, L'lanziva A, Kariuki S, Desai M, Webster J., 2016. User and Provider Acceptability of Intermittent Screening and Treatment and Intermittent Preventive

Treatment with Dihydroartemisinin-Piperaquine to Prevent Malaria in Pregnancy in Western Kenya. *PLoS One 11*

53. Hill J, Landuwulang CUR, Ansariadi, Hoyt J, Burdam FH, Bonsapia I, Syafruddin D, Poespoprodjo JR, ter Kuile FO, Ahmed R, Webster J., 2018. Evaluation of the national policy of single screening and treatment for the prevention of malaria in pregnancy in two districts in Eastern Indonesia: Health provider perceptions. *Malar J 17*: 1–12

54. Hoyt J, Landuwulang CUR, Ansariadi, Ahmed R, Burdam FH, Bonsapia I, Poespoprodjo JR, Syafruddin D, ter Kuile FO, Webster J, Hill J., 2018. Intermittent screening and treatment or intermittent preventive treatment compared to current policy of single screening and treatment for the prevention of malaria in pregnancy in Eastern Indonesia: acceptability among health providers and pregnant women. *Malar J 17*

Supplementary Table 3: Data extraction from included studies

Study. 1 Halliday et al. (2014)

**Study characteristics**	
METHODS	
Study dates	01/2010 - 03/2012
Location	Kenya: Kwale - Mkongani and Shimba Hills; Msambweni - Lunga and Mwereni
Peak transmission season	Perennial: following rainy seasons which are April - July; September - November
Baseline transmission intensity	Moderate, baseline estimates 9-24% prevalence; baseline survey of study population 13%
Parasite species	*Plasmodium falciparum*
Vector species	*A. funestus, A. gambiae s.l.*
Study design	Factorial Cluster-randomized controlled trial
Statistical power calculation	The sample size required for a power of 80% at a two-sided significance level of 5%, was a total of 27 schools in each arm with 50 children per school to detect a 25% reduction in the prevalence of anaemia, which was the primary outcome
Clusters	Unit of randomization: School<br>Number of clusters selected: 101<br>Number of clusters analyzed: 101<br>Average cluster size: Baseline: Control - n= 50 clusters; mean (SD) = 47.5 (6.9), range 16-55; Intervention - n = 51; mean (SD) 47.1 (6.1), range 26-60; Follow-up 12 months - Control mean = 43 (6.6) [15-53]; Intervention mean 45.1 (4.4) [34-55]; Follow-up 24 months Control mean 40.5 (6.3) [15,49]; Intervention mean 42.6 (4.9) [33,55]
PARTICIPANTS	
Targeted population	Total: 13527<br>Intervention: 3850 randomized for malaria intervention<br>Comparison: 3487 randomized for participation in malaria control arm
Participant characteristics	School-aged children, 5 to 20 years
INTERVENTION	
Intervention	School based intermittent screening and treatment
Comparator	No intervention
Background interventions	Lymphatic filariasis treatment with albendazole and praziquantel<br>Literary and educational assessments<br>Bed nets, high coverage though not specified
Diagnostics	Microscopy: Thick and thin blood films were stained with Giemsa, asexual parasites were counted against 200 white blood cells (WBCs); read independently and blinded by two experts<br>RDT: ParaCheck-Pf device, Orchid Biomedical Systems
Drug and manufacturer	artemether-lumefantrine (AL); Coartem, Novartis
Dosage	6 doses over 3 days in four categories (15 kg, 15–24.9 kg, 25–34.9 kg, and 35 kg). AL was given at a dose of 20/120 mg to children ,15 kg, 40/240 mg to children 15–24.9 kg, 60/360 mg to children 25–34.9 kg, and 80/480 mg to those who weighed >35 kg.

Number of rounds per season or year	Five rounds of screening and treatment - March 2010, July 2010, September 2010, March 2011, October 2011; Follow up surveys in March 2011 and March 2012.
TTaT interval	Accordance with school terms; 3-6 months
Duration of the intervention	2 years
TTaT adherence	During the 24 months of intervention, an average of 2,340 children (88.4% of eligible study children) in the 51 intervention schools were screened at each visit. There was an apparent decline in full supervision (a proxy for compliance) with time, falling from 96.9% at the first round to 81.7% at the fifth round.
OUTCOMES	
Prevalence of infection among those targeted by the intervention	Measurement: At 12-months follow-up, 2,148 children in the control schools and 2,298 in the intervention schools provided a finger-prick blood sample for Hb assessment, and at 24 months 2,027 and 2,174 children provided finger-prick samples in the control and intervention groups, respectively.<br>Timepoints: 12 -, and 24-months follow-up<br>Sample size: 12-months: 2298 (intervention), 2148 (control); 24-months: 2174 (intervention); 2027(control)
Adverse Events	Measurement: active surveillance for 48 hours and passive surveillance up to 28 days<br>Timepoints: up to 28 days<br>Sample size: 2030 intervention arm, control arm not reported
Severe Adverse Events	Measurement: death<br>Timepoint: 24 months<br>Sample size: 12-months: 2298 (intervention), 2148 (control); 24-months: 2174 (intervention); 2027(control)

Study 1. Halliday et al. (2014)

**Risk of bias**		
Outcome: Prevalence of infection in the group targeted by the intervention		
**Domain**	**Author's judgement**	**Justification**
D1a: Randomization process	Low risk	"Schools were randomized to one of four groups, receiving either: (i) IST alone; (ii) the literacy intervention alone; (iii) both interventions combined; or (iv) control group where neither intervention was implemented…,The 101 schools were randomized in two stages with each stage conducted during a public ceremony. Schools are aggregated into sets of between three and six closely located schools, which regularly meet and share information, supported by a Ministry of Education Teacher Advisory Centre tutor. The IST intervention was randomly allocated at the level of the school, with the 101 schools re-stratified by (i) literacy intervention group assignment and (ii) quintiles of average school exam scores.)."<br><br>The geographical heterogeneity observed in the prevalence of P. falciparum infection is likely to reflect a complexity of factors that influence vector

		distribution and density as well as vector–human contact and human infection.
D1b: Timing of identification or recruitment of participants	Low risk	No randomization occurred at the school level and students were given the assigned intervention of their school.
D2: Deviations from the intended interventions	Low risk	The study was not blinded.<br>No indication of deviation from intended intervention 1. it was not possible to blind the parents, participants, or field officers delivering the IST intervention to experimental assignment, which could have led to a possible ‘‘John Henry’’ effect whereby children in the control group adjust their behavior as they know they are not receiving the intervention, for example in risk aversion and treatment seeking behavior. 2) Second, study children's access to alternative malaria treatments outside of the school-based IST rounds was not monitored during the two years of the trial. 3) the lack of multiple testing adjustments may have increased the possibility of type 1 error, and results should be interpreted in light of this possible error, but it is unlikely to have masked a beneficial effect of IST.
D3: Missing outcome data	Low risk	Of the 5,233 children enrolled initially, 4,446 (85.0%) were included in the 12-month follow-up health survey and 4,201 (80.3%) were included in the 24-month health survey. At 12 and 24 months, children lost to follow-up across both study arms were largely similar to children followed up.<br><br>Lowest cluster range was 26 at any timepoint (figure 3)
D4: Measurement of the outcome	Low risk	The analyses described here correspond to a pre-specified statistical analysis plan, approved by both the data monitoring committee and trial steering committee before any data were examined.
D5: Selection of the reported result	Low risk	Reported primary and secondary outcomes are the same as reported on clinicaltrials.gov.<br><br>The primary pre-specified analysis adjusted for age (as a continuous variable), sex, and the baseline measure of the outcome, except for baseline P. falciparum, which was not measured in the control schools.

**Risk of bias**		
Outcome: Adverse Events		
Domain	**Author's judgement**	**Justification**
D1a: Randomization process	Low risk	"Schools were randomised to one of four groups, receiving either: (i) IST alone; (ii) the literacy intervention alone; (iii) both interventions combined;

		or (iv) control group where neither intervention was implemented…,The 101 schools were randomized in two stages with each stage conducted during a public ceremony. Schools are aggregated into sets of between three and six closely located schools, which regularly meet and share information, supported by a Ministry of Education Teacher Advisory Centre tutor. The IST intervention was randomly allocated at the level of the school, with the 101 schools re-stratified by (i) literacy intervention group assignment and (ii) quintiles of average school exam scores.).”<br><br>The geographical heterogeneity observed in the prevalence of P. falciparum infection is likely to reflect a complexity of factors that influence vector distribution and density as well as vector–human contact and human infection.
D1b: Timing of identification or recruitment of participants	Low risk	No randomization occurred at the school level and students were given the assigned intervention of their school.
D2: Deviations from the intended interventions	Low risk	The study was not blinded.<br>No indication of deviation from intended intervention
D3: Missing outcome data	Some Concerns	Adverse events were monitored by the study team for 24 hours after each treatment, and a further 28 days thereafter using a passive surveillance system in schools.<br>Adverse events not monitored in control arm
D4: Measurement of the outcome	Low risk	Adverse events were monitored using passive surveillance in the schools for up to 24 months follow-up period.
D5: Selection of the reported result	Low risk	Adverse events were monitored by the study team for 24 hours after each treatment, and a further 28 days thereafter using a passive surveillance system in schools. Travel costs were reimbursed, and treatment charges waived. Adverse experiences were monitored until the event was cured or had stabilised. Agranulocytosis and hepatotoxicity not monitored.

Risk of bias		
Outcome: Severe Adverse Events		
Domain	**Author’s judgement**	**Justification**
D1a: Randomization process	Low risk	“Schools were randomized to one of four groups, receiving either: (i) IST alone; (ii) the literacy intervention alone; (iii) both interventions combined; or (iv) control group where neither intervention was implemented…,The 101 schools were randomized in two stages with each stage conducted during a public ceremony. Schools are aggregated into sets of between three and six closely located schools, which regularly meet and share information, supported by a

		Ministry of Education Teacher Advisory Centre tutor. The IST intervention was randomly allocated at the level of the school, with the 101 schools re-stratified by (i) literacy intervention group assignment and (ii) quintiles of average school exam scores.).”<br><br>The geographical heterogeneity observed in the prevalence of P. falciparum infection is likely to reflect a complexity of factors that influence vector distribution and density as well as vector–human contact and human infection.
D1b: Timing of identification or recruitment of participants	Low risk	No randomization occurred at the school level and students were given the assigned intervention of their school.
D2: Deviations from the intended interventions	Low risk	The study was not blinded.<br>No indication of deviation from intended intervention
D3: Missing outcome data	Low risk	During the 24 months of follow-up, 11 children died: five in the intervention group and six in the control group. Cause of death was investigated and included yellow fever, heart defect, leukemia, drowning, trauma, pneumonia, and pediatric HIV.
D4: Measurement of the outcome	Low risk	Mortality was monitored for study participants during the 24-month follow-up period.
D5: Selection of the reported result	Low risk	Reported primary and secondary outcomes are the same as reported on clinicaltrials.gov.

Study 2. Baiden et al. (2016)

**Study characteristics**	
METHODS	
Study dates	02/2009-07/2012
Location	Ghana: the forest-savannah transition zone
Peak transmission season	Perennial
Baseline transmission intensity	High: (EIR) estimated at 231–269 infective bites per person per year
Parasite species	*Plasmodium falciparum*
Vector species	*Anopheles funestus and Anopheles gambiae*
Study design	Factorial Cluster-randomized controlled trial
Statistical power calculation	To reach 80% power at 95% significance and detect a 20% difference in the incidence of malaria between the two comparison groups at least 30 clusters (15 per arm) and 200 person-years at risk (PYAR) per cluster. This was based on<br>the following expectations: (1) Incidence of malaria in the CJ arm would be 0.8 episode/ 1 per child per year (the rate observed in a group of < 24-month-old children in Navrongo, Ghana)

Clusters	Unit of randomization: Health center; Thirty-two health centers were stratified by districts, ranked by the number of Diptheria-Pertussis-Tetanus1 (DPT1) vaccinations given in the facility in the preceding year and paired according to the rank within each district<br>Number of clusters selected: 32<br>Number of clusters analyzed: 32<br>Average cluster size: 95 participants
PARTICIPANTS	
Targeted population	Total: 3046<br>Intervention: 1585 in the RDT arm<br>Comparison: 1579 clinical judgement arm
Participant characteristics	Children < 24 months
INTERVENTION	
Intervention	RDT-based testing w/fever screening and treatment
Comparator	Standard of care/ clinical judgement
Background interventions	Bed nets: 74.4% in intervention; 79% in CJ group<br>All providers given refresher training in IMCI management of febrile illnesses every 4-6months<br>Febrile illness and morbidity questionnaires by field investigators
Diagnostics	Two brands of mRDTs procured through the Ghana National Malaria Control Programme, CareStart™ and First Response™<br>Microscopy: blood smears read independently by two expert microscopists at the reference laboratory at the Kintampo Health Research Centre (KHRC)
Drug and manufacturer	Nationally approved first line ACTs: artesunate-amodiaquine, artemether-lumefantrine and dihydroartemisinin-piperaquine
Dosage	Not specified
Number of rounds per season or year	One round for children who reported with fever; time to first episode of febrile illness
TTaT interval	n/a
Duration of the intervention	2 years
TTaT adherence	As a deviation from the study protocol, 8 out of 5799 (0.14%) cases that presented to CJ-arm facilities had RDT performed.
OUTCOMES	
Incidence of malaria infection (community level)	Measurement: survival time to malaria after the first episode of a febrile illness and person-time to first episode of febrile illness<br>Timepoints: 24-months follow-up<br>Sample size: 1527 in the RDT arm and 1519 in the CJ- arm
Severe Adverse Events	Measurement: death<br>Timepoint: 24 months<br>Sample size: Control = 1498 RDT arm = 1512

Study 2. Baiden et al. (2016)

Risk of bias		
Outcome: Incidence of malaria infection		
Domain	Author's judgement	Justification
D1a: Randomization process	Low risk	"Thirty-two health centers were stratified by districts, ranked by the number of Diptheria-Pertussis-Tetanus1 (DPT1) vaccinations given in the facility in the preceding year and paired according to the rank within each district. Within each pair of health facilities, one was allocated randomly to implement test-based (RDT-arm) management of malaria, while the other health facility within the pair was allocated to implement the clinical judgement (CJ arm) approach."<br>No significant differences in the characteristics and comparability of children enrolled into the two arms of the study.
D1b: Timing of identification or recruitment of participants	Low risk	Investigators enumerated all households that were located within 2km radius of the selected health centers and enrolled one hundred children per health facility who met the inclusion criteria; they enrolled children living closest to the selected health centers and extended outwards circumferentially until the target of 100 children was achieved.<br>No significant differences in the characteristics and comparability of children enrolled into the two arms of the study.
D2: Deviations from the intended interventions	Low risk	Caregivers had to provide consent to participation and received assistance for enrollment in the national insurance scheme as well home visits from field workers.<br>All children received clinical assessment for malaria appropriately that was not obviously linked to the intervention assignment from a service delivery standpoint. Children in the RDT group were tested using RDTs from the NMCP and were also treated for any concurrent illnesses according to IMCI guidelines. In the control group children who presented with fever were not tested for malaria. The treatment for malaria in these facilities was presumptive and based on presenting clinical symptoms and signs. The treatment of all other ailments were based on the IMCI guidelines.<br>The fieldworkers stationed in each (intervention and control) health facility helped to ensure compliance with the protocol by reminding attending clinicians about the protocol of the study, as applicable to the particular health centre. This was reinforced during the 4–6 monthly training sessions that were held for health workers in both intervention and control facilities.

		A multilevel Poisson model assuming unstructured covariance was used to estimate the incidence of malaria over the 24-month period to account for repeat episodes of malaria within a child, and clustering by health facility.
D3: Missing outcome data	Low risk	In control and intervention group 405 and 355 individuals were not seen at least every 100 days post follow-up initiation.<br>Per protocol analysis produced similar results as the ITT analysis
D4: Measurement of the outcome	Low risk	Data were double-entered using FoxPro version 9. The analysis was conducted using STATA version 12.
D5: Selection of the reported result	High risk	A Data Safety Monitoring Board (DSMB) periodically reviewed safety parameters and incidence of malaria and anaemia. The DSMB also approved the final analysis plan.<br>Conducted a multi-level Poisson to calculate incidence and rate ratios for comparison in study arms, but did not perform a generalized model accounting for potential demographics and confounders to assess risk of malaria infection in study arms.

**Risk of bias**		
Outcome: Serious Adverse Events		
Domain	Author's judgement	Justification
D1a: Randomization process	Low risk	"Thirty-two health centers were stratified by districts, ranked by the number of Diptheria-Pertussis-Tetanus1 (DPT1) vaccinations given in the facility in the preceding year and paired according to the rank within each district. Within each pair of health facilities, one was allocated randomly to implement test-based (RDT-arm) management of malaria, while the other health facility within the pair was allocated to implement the clinical judgement (CJ arm) approach."<br>No significant differences in the characteristics and comparability of children enrolled into the two arms of the study.
D1b: Timing of identification or recruitment of participants	Low risk	Investigators enumerated all households that were located within 2km radius of the selected health centers and enrolled one hundred children per health facility who met the inclusion criteria; they enrolled children living closest to the selected health centers and extended outwards circumferentially until the target of 100 children was achieved.<br>No significant differences in the characteristics and comparability of children enrolled into the two arms of the study.
D2: Deviations from the intended interventions	Low risk	Caregivers had to provide consent to participation and received assistance for enrollment in the national

		insurance scheme as well home visits from field workers.<br>All children received clinical assessment for malaria appropriately that was not obviously linked to the intervention assignment from a service delivery standpoint. Children in the RDT group were tested using RDTs from the NMCP and were also treated for any concurrent illnesses according to IMCI guidelines. In the control group children who presented with fever were not tested for malaria. The treatment for malaria in these facilities was presumptive and based on presenting clinical symptoms and signs. The treatment of all other ailments were based on the IMCI guidelines.<br>The fieldworkers stationed in each (intervention and control) health facility helped to ensure compliance with the protocol by reminding attending clinicians about the protocol of the study, as applicable to the particular health centre. This was reinforced during the 4–6 monthly training sessions that were held for health workers in both intervention and control facilities.<br>A multilevel Poisson model assuming unstructured covariance was used to estimate the incidence of malaria over the 24-month period to account for repeat episodes of malaria within a child, and clustering by health facility.
D3: Missing outcome data	Low risk	In control and intervention group 405 and 355 individuals were not seen at least every 100 days post follow-up initiation. Deaths were recorded in both the clinical judgement and intervention arms
D4: Measurement of the outcome	Low risk	Data were double-entered using FoxPro version 9. The analysis was conducted using STATA version 12.
D5: Selection of the reported result	High risk	Although mortality was measured in the study, it was assumed a priori that the number of deaths would not be sufficient to detect any significant difference between the two arms.

Study 3. Cohee et al. (2021)

**Study characteristics**	
METHODS	
Study dates	2015
Location	Southern Malawi: Maseya, Makhuwira, Bvumbwe, Ngowe
Peak transmission season	Rainy season (April–May) and Dry season (September–October)
Baseline transmission intensity	Moderate<br>Maseya and Makjuwira: >40% parasite prevalence in school children<br>Bvumbwe and Ngowe : lower, seasonally varied transmission (> two-fold seasonal prevalence difference)

Parasite species	*Plasmodium falciparum*
Vector species	Not specified
Study design	Cohort Study
Statistical power calculation	n/a
Cohorts	Unit of randomization: Grade/Classroom in school by village<br>Number of participants by group: Fifteen students per grade-level (grade 1–8)<br>Number of cohorts assessed: 2 cohorts, rainy season and dry season
PARTICIPANTS	
Targeted population	Total: 960<br>Before: 786<br>After: 616
Participant characteristics	School-aged children, 5 to 15 years
INTERVENTION	
Intervention	School based intermittent screening and treatment
Comparator	Pre - intervention baseline of cohort
Background interventions	Bed nets: 47% (reported bed net use the previous night)
Diagnostics	qRT-PCR: finger prick; filter paper (Whatmann #3) dried blood spots and whole blood in RNA preservative<br>RDT from Standard of Care - histidine rich protein 2 RDT from Paracheck Orchid Biomedical Systems, Goa, India or SD Bioline, Standard Diagnostics Inc.,Suwon City, Republic of Korea
Drug and manufacturer	artemether-lumefantrine (AL); Novartis Pharma AG or Ajanta Pharma Ltd.
Dosage	weight-based
Number of rounds per season or year	one round per cohort
TTaT interval	n/a; follow-up at 1, 2 and 6 weeks
Duration of the intervention	6 weeks
TTaT adherence	Not specified
OUTCOMES	
Prevalence of infection among those targeted by the intervention	Measurement: At baseline and all follow-up visits, finger-prick blood was obtained for molecular detection of any-stage parasites and gametocytes<br>Timepoints: baseline, 1-, 2-, 6-weeks<br>Sample size: 786 ‘before’; 616 ‘after’

Supplementary Figure 1: Risk of Bias Assessment for outcomes in cluster randomized control trials by domains

Study	Outcome	D1a	D1b	D2	D3	D4	D5	Overall
Baiden et al. (2016)	Incidence of malaria infection	+	+	+	+	+	-	-
Halliday et al. (2014)	Prevalence of infection	+	+	+	+	+	+	+
Halliday et al. (2014)	Adverse Events	+	+	+	!	+	+	!
Baiden et al. (2016)	Severe Adverse Events	+	+	+	+	+	+	+
Halliday et al. (2014)	Severe Adverse Events	+	+	+	+	+	+	+

Domains

D1a	Randomization process
D1b	Timing of identification or recruitment of participants
D2	Deviations from the intended interventions
D3	Missing outcome data
D4	Measurement of the outcome
D5	Selection of the reported result

Judgement

+	Low risk
!	Some concerns
-	High risk

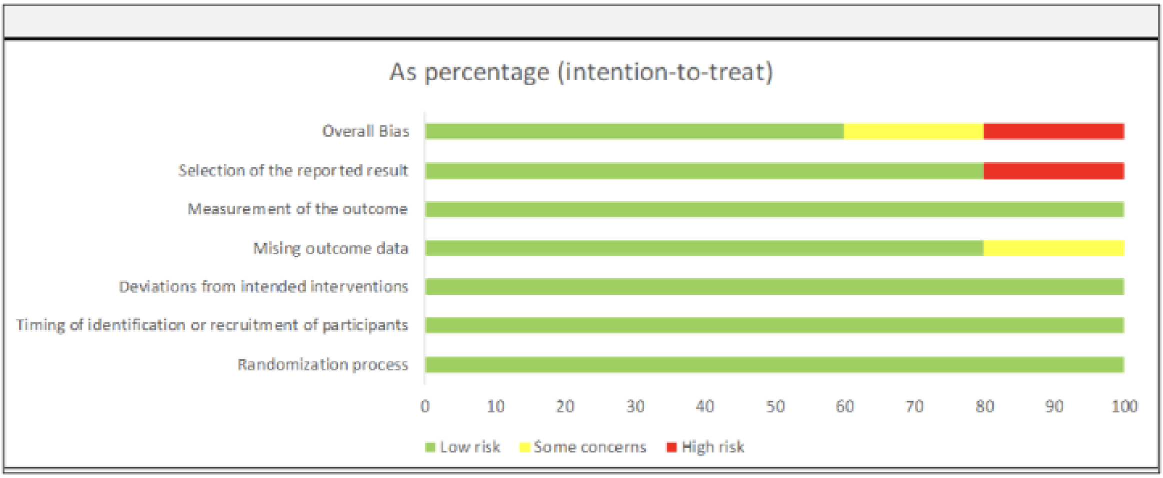

Supplementary Figure 2: Risk of Bias summary for controlled before and after cohort study

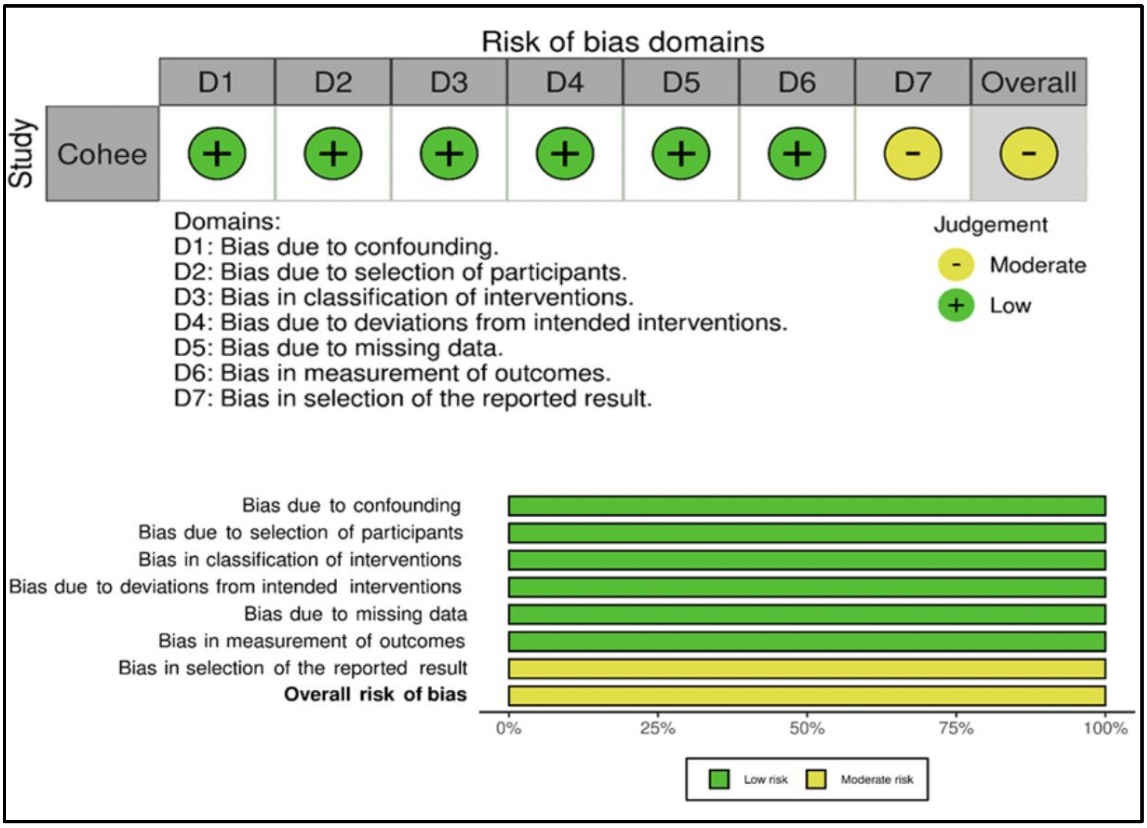

Supplement: Supplemental Materials [file tpmd230097.SD1.pdf]
